# Folate deficiency may increase the risk for elevated TSH in patients with type 2 diabetes mellitus

**DOI:** 10.1186/s12902-023-01422-2

**Published:** 2023-08-11

**Authors:** Lin Lin, Yushan Du, Guanyu Niu, Shuangbo Xia, Jufen Liu

**Affiliations:** 1 Department of Endocrinology, Beijing Chao-Yang Hospital, Capital Medical University, Beijing, China; 2 Institute of Reproductive and Child Health / National Health Commission Key Laboratory of Reproductive Health, Peking University, Beijing, China; 3 Department of Epidemiology and Biostatistics, School of Public Health, Peking University, Beijing, China; 4 Department of Epidemiology and Health Statistics, School of Public Health, Capital Medical University, Beijing, China

**Keywords:** Serum folate, Thyroid dysfunction, Thyroid stimulating hormone, Type 2 diabetes mellitus

## Abstract

**Background:**

Type 2 diabetes mellitus (T2DM) and thyroid dysfunction (TD) are two common chronic endocrine disorders that often coexist. Folate deficiency has been reported to be related with the onset and development of T2DM. However, the relationship between folate deficiency and TD remains unclear. This study aims to investigate the association of serum folate with TD in patients with T2DM.

**Methods:**

The study used data on 268 inpatients with T2DM in the Beijing Chao-yang Hospital, Capital Medical University from October 2020 to February 2021. Thyroid stimulating hormone (TSH), free triiodothyronine (FT3), free thyroxine (FT4), and serum folate were measured with chemiluminescence immunoassay (CLIA), and folate deficiency was defined as a serum folate concentration < 4.4 ng/mL. Ordinary least squares regression models were used to assess the association of serum folate with TSH concentration. Multivariable logistic regression models were performed to explore the correlation of folate deficiency and the risk for elevated TSH.

**Results:**

15.3% of T2DM patients had TD. Among those patients with TD, 80.5% had elevated TSH. Compared with the normal-TSH and low-TSH groups, the prevalence of folate deficiency was significantly higher in the elevated-TSH group (*P* < 0.001). Serum folate level was negatively associated with TSH (β=-0.062, 95%CI: -0.112, -0.012). Folate deficiency was associated with the higher risk for elevated TSH in patients with T2DM (OR = 8.562, 95%CI: 3.108, 23.588).

**Conclusions:**

A low serum folate concentration was significantly associated with a higher risk for elevated TSH among T2DM patients.

## Introduction

With the accelerated speedup of ageing and urbanization, and concurrent increases in obesity and unhealthy lifestyle, Type 2 diabetes mellitus (T2DM) has become a global public health concern. T2DM is a risk factor for cardiovascular disease (CVD), and CVD is the leading cause of increased morbidity and mortality in T2DM patients [1]. It has been estimated that CVD costs contributed between 20% and 49% of the total direct costs of treating T2DM [2]. The prevalence of T2DM in China was high and brought an enormous health burden. It has been estimated that there were about 20.8 million people in China who had T2DM in the year 2000, and the number of patients with T2DM will double in 2030 [3]. The direct medical costs of T2DM and its complications in China were estimated to be 47.2 billion USD in 2030 [4].

Thyroid dysfunction (TD) is another most common endocrinopathy. The prevalence of TD was significantly higher in patients with T2DM than those without T2DM [5]. Mounting evidence has proved that TD and T2DM often coexist, and are closely linked. For instance, some antidiabetic drugs (e.g. metformin, sulfonylurea, and thiazolidinediones) affect thyroid function and influence the levels of thyroid hormones by impacting the hypothalamus-pituitary-thyroid (HPT) axis, and thyroid hormone could affect glucose homeostasis by impacting the development and function of pancreatic β-cell [6, 7]. Recent evidence has indicated that TD may be associated with the risk for the development of diabetic complications [8, 9]. And thyroid hormones play an important role in maintaining cardiovascular homeostasis, the onset and development of TD could further increased cardiovascular risk in patients with T2DM [10]. Following the mandatory universal salt iodization, subclinical hypothyroidism has become the most commonly observed TD subtype in China, which may attributable to elevated serum thyroid stimulating hormone (TSH) level [11]. Subclinical hypothyroidism and elevated TSH concentration is associated with an increased risk for CVD and all-cause mortality, particularly in individuals with high CVD risk (e.g. T2DM patients) [12, 13]. Therefore, the early prevention and screening of TD in T2DM patients may be important to reduce the burden of disease.

Folate plays an important role in DNA synthesis, amino acid homeostasis, antioxidant generation, and epigenetic regulation repair, and adequate folate intake is essential for maintaining the metabolism homeostasis [14]. Evidence has indicated that folate deficiency is associated with the development of hyperhomocysteinemia (HHcy) among T2DM patients [15]. Moreover, a study found that hypothyroid patients had elevated concentration of Hcy and a lower level of folate [16]. TD and T2DM are closely linked, and increased the risk for cardiovascular disease (CVD). Clinical trials have revealed that folate supplementation could reduce the oxidative stress and DNA damages in patients with T2DM, and may have a potential effect on the prevention of future cardiovascular complications [17, 18]. However, the relationship between folate and TD were still unclear in patients with T2DM. Therefore, the present study aimed to investigate the association of serum folate level and TD in patients with T2DM.

## Materials and methods

### Study participants

A total of 299 patients with T2DM were recruited from Beijing Chao-yang Hospital, Capital Medical University from October 2020 to February 2021. The diagnosis of T2DM was performed based on the World Health Organization (WHO) criterion which was published in 1999 [19]. The present analysis excluded patients with invalid data for TSH (n = 9), serum folate (n = 10), vitamin B_12_ (n = 12). The final sample included 268 patients.

We used PASS 15.0 (NCSS, LLC, Kaysville, UT, USA) to estimate the minimum sample size. With an expected prevalence of TD in T2DM patients (23.8%) based on the previous study [20], for α = 0.05 and β = 0.20, the sample size of 208 patients allowed for the calculation of a 95% confidence interval (CI) with a precision of +/- 12%. Therefore, the sample size of the included study participants can meet the need of statistical power.

### Clinical data collection

Demographics, clinical parameters, and medical history were collected through the electronic medical record of Beijing Chao-Yang Hospital, including age, gender, weight, height, waist circumference, systolic blood pressure (SBP), diastolic blood pressure, high-density lipoprotein cholesterol (HDL-C), total cholesterol (TC), triglyceride (TG), homocysteine (Hcy), folate, vitminB12, TSH, free triiodothyronine (FT3), free thyroxine (FT4). Blood samples were collected after an overnight fast. Blood biochemical indices were examined using Siemens Advia 2400 automatic analyzer. TSH, FT3, FT4, serum folate, and serum vitminB12 were determined by chemiluminescence immunoassay (CLIA).

### Definitions of variables

The reference range for serum TSH is 0.45 to 4.50 µU per mL, the reference range for serum FT3 is 2.3 pmol/L to 6.3 pmol/L, and the reference range for serum FT4 is 10.3 pmol/L to 24.5 pmol/L [21]. Low TSH was defined as a TSH < 0.45 mU/L, elevated TSH was defined as a TSH > 4.50 mU/L. Hyperthyroidism was defined as TSH < 0.45mU/L, when FT3 and/or FT4 above their reference ranges. Hypothyroidism was defined as TSH > 4.50mIU/L when FT3 and/or FT4 below their reference ranges. Subclinical hyperthyroidism was defined as TSH < 0.45mU/L when FT3 and FT4 within their reference ranges. Subclinical hypothyroidism was defined as TSH > 4.50mU/L when FT3 and FT4 within their reference ranges. Folate deficiency was defined as a serum folate concentration below 4.4 ng/mL (< 10 nmol/L) based on WHO cut-off values [22]. Body mass index (BMI) was calculated as weight in kilograms divided by height in meters squared, and was categorized as underweight/ normal weight (< 24 kg/m^2^), overweight (24 ≤ to < 28 kg/m^2^), obese (≥ 28 kg/m^2^) [23].

### Statistical analysis

Continuous variables are presented as medians with IQRs. Categorical variables are presented as frequencies and percentages. The Kruskal Wallis test was used to compare variables with skewed distributions, and categorical variables were tested with the Fisher’s exact test. Ordinary least squares (OLS) regression models were used to examine the association with concentration of folate, folate deficiency and concentration of TSH. And we further used logistic regression models to examine the association with folate deficiency and the risk for elevated TSH. The multivariate models were adjusted for the continuous variables (i.e. age, Vitamin B12, Hcy, BMI, HDL-C, TG, SBP, and DBP), and the categorical variable (i.e. sex).

The software Stata version 15 for Windows (Stata Corp, College Station, TX, USA) was used to perform statistical analyses, and a two-sided *P* < 0.05 was considered statistically significant.

### Ethical approval and consent

This study was carried out in compliance with the International Ethical Guidelines on Biomedical Research Involving Human Subjects and was approved by the hospital ethics committee of Affiliated Beijing Chao-Yang Hospital of Capital Medical University.

## Results

### Basic characteristics of the study participants

Patients’ characteristics are shown in Table 1. Of the 268 patients included, 166 (61.9%) were men, and the median (IQR) age was 56.0 years (IQR 19.5). There were statistically significant differences among the three groups (normal TSH, elevated TSH, and low TSH) in folate deficiency (*P* < 0.01). And the elevated TSH group had the lowest serum folate level.

**Table 1:** Characteristics for T2DM patients, (N = 268)

	Total participants (n = 268)	Normal TSH ^a^ (n = 227)	Elevated TSH ^b^ (n = 33)	Low TSH ^c^ (n = 8)	*P*
Age, Median (IQR)	56.0 (19.5)	55.0 (19.0)	60.0 (16.0)	63 (18.0)	0.182 ^e^
Sex, n (%)					
Men	166 (61.9)	148 (65.2)	15 (45.4)	3 (37.5)	0.031 ^f^
Women	102 (38.1)	79 (34.8)	18 (54.6)	5 (62.5)	
Folate [(ng/mL), Median (IQR)]	9.4 (8.3)	9.4 (8.2)	7.9 (10.1)	8.2 (8.4)	0.247 ^e^
Folate deficiency ^d^					
No	240 (89.6)	211 (96.0)	22 (66.7)	7 (87.5)	< 0.001 ^f^
Yes	28 (10.4)	16 (7.0)	11 (33.3)	1 (12.5)	
Vitamin B_12_ [(pg/mL), Median (IQR)]	544.0 (436.0)	539.0 (421.0)	748.0 (588.4)	466.1 (398.0)	0.036 ^e^
Hcy [(µmol/L), Median (IQR)]	11.0 (3.5)	11.0 (4.0)	11.0 (2.0)	12.0 (2.5)	0.747 ^e^
BMI					
Underweight/ normal weight	66 (24.6)	59 (26.0)	7 (21.2)	0 (0.0)	0.167 ^f^
Overweight	124 (46.3)	99 (43.6)	18 (54.6)	7 (87.5)	
Obese	78 (29.1)	69 (30.4)	8 (24.2)	1 (12.5)	
HDL-C [(mmol/L), Median (IQR)]	1.1 (0.5)	1.1 (0.5)	1.1 (0.6)	1.3 (0.6)	0.714 ^e^
TG [(mmol/L), Median (IQR)]	1.4 (0.8)	1.4 (0.9)	1.4 (1.0)	1.2 (0.8)	0.306 ^e^
SBP [mmHg, Median (IQR)]	129.7 (19.0)	129.4 (20.0)	129.0 (16.0)	140.0 (23.5)	0.159 ^e^
DBP [mmHg, Median (IQR)]	76.0 (13.0)	76.0 (13.0)	74.0 (8.0)	84.5 (26.5)	0.265 ^e^

Note

^a^ Normal TSH was define as TSH concentrations within its reference range (0.45 to 4.50 mU/L);

^b^ Elevated TSH was define as TSH concentrations > 4.50 mU/L;

^c^ Low TSH was define as TSH concentrations > 0.45 mU/L;

^d^ Folate deficiency was defined as serum folate concentrations < 4.4 ng/mL;

^e^ Kruskal–Wallis test; ^f^ Fisher’s exact test;

Abbreviations: TSH, thyroid-stimulating hormone; IQR, interquartile range; Hcy, homocysteine; BMI: body mass index; HDL-C, high density liptein cholesterol; TG, Triglyceride; SBP, systolic blood pressure; DBP, diastolic blood pressure

The details of patients with thyroid dysfunction are shown in Table 2. The prevalence of TD was 15.3%. Most of the patients with TD had subclinical hypothyroidism. Specifically, the prevalence of subclinical hypothyroidism, hypothyroidism, subclinical hyperthyroidism, and hyperthyroidism were 11.9%, 0.4%, 2.2%, 0.7%, respectively. 10.4% of T2DM patients had folate deficiency, and the prevalence folate deficiency in patients with subclinical hypothyroidism was 34.3%.

**Table 2:** Details of thyroid dysfunction in T2DM patients, (N = 268)

Thyroid dysfunction, n (%)	Total participants (n = 268)	Folate deficiency	
		Yes (n = 28)	No (n = 240)
With thyroid dysfunction	41 (15.3)	12 (42.9)	29 (12.1)
Subclinical hypothyroidism	32 (78.1)	11 (91.7)	21(72.4)
Hypothyroidism	1 (2.4)	0 (0.0)	1 (3.5)
Subclinical hyperthyroidism	6 (14.6)	0 (0.0)	6 (20.7)
Hyperthyroidism	2 (4.9)	1 (8.3)	1 (3.4)
Without thyroid dysfunction	227 (84.7)	16 (57.1)	211 (87.9)

### Association of folate level with TSH in patients with T2DM

The results of the OLS regression modeling on the associations between folate and TSH are presented in Table 3. After controlling for all covariates, higher folate concentration was associated with a lower TSH level (β = -0.062, 95% CI: -0.112, -0.012). T2DM patients had folate deficiency had an elevated TSH level with a significant coefficient of 2.444 (95% CI: 1.535, 3.352) than those without folate deficiency.

**Table 3:** Associations of folate with TSH among T2DM patients, (N = 268)

Variable	Unadjusted β(95% CI)	Adjusted β(95% CI)
Per 1 ng/mL increase in serum folate	-0.446 (-0.880, -0.001)	-0.062 (-0.112, -0.012)
Folate deficiency ^b^		
No	Ref.	Ref.
Yes	2.384 (1.513, 3.256)	2.444 (1.535, 3.352)

Note

^a^ Adjusted for age, sex, Vitamin B_12_, Hcy, BMI, HDL-C, TG, SBP, DBP;

^b^ Folate deficiency was defined as serum folate concentrations < 4.4 ng/mL

Abbreviations: TSH, thyroid-stimulating hormone; CI, confidence interval; Hcy, homocysteine; BMI: body mass index; HDL-C, high density liptein cholesterol; TG, Triglyceride; SBP, systolic blood pressure; DBP, diastolic blood pressure

After adjustment for multiple potential confounders, including age, sex, Vitamin B12, Hcy, BMI, HDL-C, TG, SBP, and DBP, folate deficiency was associated with a higher risk for elevated TSH among T2DM patients (odds ratio = 8.562, 95% CI: 3.108, 23.588; Table 4).

**Table 4:** Associations of folate deficiency with evaluated TSH among T2DM patients, (N = 268)

Variable	Unadjusted OR (95% CI)	Adjusted ^a^ OR (95% CI)
Folate deficiency ^b^		
No	Ref.	Ref.
Yes	6.412 (2.670, 15.397)	8.562 (3.108, 23.588)

Note

^a^ Adjusted for age, sex, Vitamin B_12_, Hcy, BMI, HDL-C, TG, SBP, DBP;

^b^ Folate deficiency was defined as serum folate concentrations < 4.4 ng/mL

Abbreviations: TSH, thyroid-stimulating hormone; OR, odds ratio; CI, confidence interval; Hcy, homocysteine; BMI: body mass index; HDL-C, high density liptein cholesterol; TG, Triglyceride; SBP, systolic blood pressure; DBP, diastolic blood pressure

## Discussion

The current study showed that folate level was correlated with TSH level in patients with T2DM. And a low serum folate concentration (< 4.4 ng/mL) was significantly associated with a higher risk for elevated TSH among T2DM patients.

Previous studies have suggested that T2DM was significantly associated with the risk for TD, which was consistent with our study [24, 25]. In our study, the prevalence of TD was 15.3% in patients with T2DM, which was lower than a multicenter cross-sectional observational study among older Chinese patients with T2DM (23.8%) [20]. The difference in prevalence rates between our study and Zhu et al. [20] may be due to the different age distributions. The overall median age (IQR) was 56.0 (19.5) years in our study; the other study included patients ≥ 60 years old. Because the metabolic function tends to decrease with age, and evidence has suggested that advanced age is a risk factor of TD [26].

We found that most of T2DM patients in our study with TD had elevated TSH. Though the mechanism underlying the association between T2DM and elevated TSH remains unclear, insulin resistance could be possibly explained the association partly. Insulin resistance may decrease the activity of type 2 deiodinase (DIO2) in thyrotrophic cells, and further inducing subclinical hypothyroidism or clinical hypothyroidism [27]. And as a thyroid growth factor, a high circulating level of insulin could directly stimulate thyroid proliferation, and increasing the risk for the formation of nodules [28]. In a T2DM rat model, the diabetic rats combined with mild hypothyroidism had a significantly lower level of oxidative stress than those diabetic rats with normal levels of thyroid hormones [29]. The finding has suggested that the elevated TSH could be a physiological adaptation of the body to against damage caused by T2DM. Taken together, evidence indicates that there may be a complex interdependent interaction between T2DM and elevated TSH. A previous study has revealed that after two decades of implementation of a mandatory universal salt iodization program in China, the prevalence of elevated TSH-related TD among Chinese adults has increased significantly [11]. Moreover, A meta-analysis has revealed that elevated TSH is associated with increased risk for diabetic complications (e.g. diabetic nephropathy, diabetic retinopathy, peripheral arterial disease, diabetic peripheral neuropathy) [30]. Therefore, in the context of China, screening thyroid function among patients with T2DM may be necessary, considering the prevalence of subclinical hypothyroidism in T2DM patients and the increased complications risk in T2DM patients with elevated TSH.

In addition, we found that folate deficiency was significantly associated with a higher risk for elevated TSH among T2DM patients. Few studies examined the association between folate and TD. And even fewer studies focused on T2DM patients and examined this association. A previous study conducted among Saudi pregnant women found a high correlation between serum concentrations of TSH and folate, and women with glycated hemoglobin (HbA1c) concentration above the reference range have a higher TSH level and a lower folate level compared with women with normal HbA1c [31]. However, another study observed a significantly positive association between the level of folate and TSH among patients with TD [32]. Although the correlation between folate concentration and TSH remains unclear, these studies suggest that folate may play a role in the development of TD. The mechanisms by which folate deficiency is associated with a higher risk for elevated TSH may be related to HHcy. Pervious interventions in patients with T2DM have found that treatments with metformin increased the risk for folate deficiency, which resulted in an elevated homocysteine concentration [33, 34]. Folate deficiency and HHcy could promote oxidative stress in patients with T2DM, and further induce thyroid dysfunctions [15, 35]. And evidence has indicated that elevated Hcy may be associated with impaired sensitivity to thyroid hormones, and the elevation of TSH concentration [36].

Our finding suggested the potential of folic acid (FA) supplements to improve the health of T2DM patient. Patients with T2DM might benefit from FA supplements directly, for evidence from randomized controlled trials has indicated that FA supplementation could improve glucose homeostasis and lowering IR potential [37, 38]. And an animal experimental study of hypothyroid female rats has found that nutrient supplement containing FA significantly reduce the TSH level, and may have effective effects on the treatment of hypothyroidism [39]. However, a controlled clinical trial found conducted in epilepsy patients with elevated TSH found that B-vitamin supplementation could reduce the Hcy level in patients, but could not improve their thyroid function [40]. Therefore, the potential benefits of FA supplements may vary in patients with different diseases. Further studies are needed to confirm the association between folate, FA and TD in T2DM patients.

Strength of our study is that we identified that serum folate level may be positively associated with the risk for subclinical hypothyroidism and clinical hypothyroidism in T2DM patients for the first time. Yet, there were several limitations. Firstly, because of the cross-sectional design, we were unable to make causal inferences about the relationship between folate deficiency and risk for TD. The sample was derived from an inpatient setting which maybe more serious compared with general population. Secondly, we are unable to consider other types of TD due to limited sample size. Further studies are needed to evaluate the effect of serum folate on other abnormal and different types of TD.

## Conclusion

In conclusion, a low serum folate level was significantly associated with a higher risk for elevated TSH in patients with T2DM. Further studies are needed to confirm and expand on whether and how folate deficiency in the pathogenesis of TD.

## Data Availability

The datasets used and/or analyzed during the current study are available from the corresponding author on reasonable request.

## List of abbreviations

T2DM: Type 2 diabetes mellitus
TD: Thyroid dysfunction
FT3: Thyroid stimulating hormone
FT4: Free triiodothyronine
CLIA: Free thyroxine
CVD: Chemiluminescence immunoassay
HPT: Cardiovascular disease
HHcy: Hypothalamus-pituitary-thyroid
SBP: Systolic blood pressure
HDL-C: High-density lipoprotein cholesterol
TC: Total cholesterol
TG: Triglyceride
Hcy: Homocysteine
OLS: Ordinary least squares
DIO2: Type 2 deiodinase
FA: Folic acid
